# Magnitude and predictors of elasticity of demand for morphine are similar in male and female rats

**DOI:** 10.3389/fnbeh.2024.1443364

**Published:** 2024-08-29

**Authors:** Andrew C. Harris, Peter Muelken, Shirelle X. Liu, John R. Smethells, Mark G. LeSage, Jonathan C. Gewirtz

**Affiliations:** ^1^Hennepin Healthcare Research Institute, Minneapolis, MN, United States; ^2^Department of Medicine, University of Minnesota, Minneapolis, MN, United States; ^3^Department of Psychology, University of Minnesota, Minneapolis, MN, United States; ^4^Department of Psychology, Arizona State University, Tempe, AZ, United States

**Keywords:** opioid use disorder, individual differences, sex differences, behavioral economics, morphine

## Abstract

**Introduction:**

Sex differences in vulnerability to opioid use disorder (OUD) have been reported in some clinical and preclinical studies, but findings are mixed and further research is needed in this area. The goal of this study was to compare elasticity of demand (reinforcement efficacy) in an i.v. morphine self-administration (SA) model in male and female rats using a translationally relevant behavioral economics approach. Rate of acquisition and predictors of individual differences in demand (e.g., cumulative morphine infusions during acquisition) were also evaluated in both sexes.

**Materials, methods, and results:**

Acquisition of morphine SA (0.4 mg/kg/infusion) under a fixed ratio (FR) 1 schedule of reinforcement was slower and infusions earned were lower in females than in males (*n* = 30–31/sex), but infusions earned did not differ between sexes during the FR 2 and FR 3 phases of acquisition. Increases in the FR response requirement across sessions during demand testing (FR 1–FR 96) resulted in a progressive reduction in morphine infusions in both sexes. Morphine consumption was well-described by an exponential demand function in both sexes and was associated with considerable individual vulnerability. There were no sex differences in elasticity of demand (rate of decline in morphine consumption with increasing price) or intensity of demand (consumption at zero price). A higher number of infusions earned during the FR 2 and FR 3 phases of acquisition and greater maximum response rates during demand testing were associated with lower demand elasticity (i.e., greater reinforcing efficacy) in both males and females, whereas other relationships were sex-specific (e.g., higher intensity of demand was associated with lower elasticity of demand in males but not in females).

**Conclusion:**

Our findings indicate similar elasticity of demand and predictors of individual differences in demand for morphine in male and female rats, although sex differences were observed in initial rate of acquisition and in some correlations between morphine SA measures. These data are consistent with findings of similar OUD vulnerability in males and females in some human and animal studies.

## Introduction

Nearly 100,000 Americans died of opioid overdose in 2022 (Spencer et al., 2024), and the opioid crisis has been declared a nationwide Public Health Emergency. Despite the devastating impact of opioids, only a minority of individuals who experiment with opioids develop opioid use disorder (OUD). Understanding factors contributing to individual differences in vulnerability to OUD may be useful for developing more effective preventions and treatments.

Sex can be an important contributor to OUD vulnerability, but the literature is mixed on whether males or females are more vulnerable. On one hand, opioids produce greater positive subjective effects (e.g., drug liking) and weaker negative subjective effects (e.g., nausea, dizziness) in men compared to women (Fillingim et al., 2005; Lopes et al., 2021; Comer et al., 2010), and prevalence of OUD and overdose has historically been higher in men (McHugh et al., 2021; Marsh et al., 2018; Huang et al., 2006; Back et al., 2010). However, the gap in OUD prevalence between sexes has been narrowing in recent years, with the misuse of several opioids (e.g., oxycodone) increasing at a greater rate in females than in males (McHugh et al., 2021; Jones et al., 2015). In addition, as has been reported for other drugs of abuse (e.g., alcohol) (Piazza et al., 1989; Towers et al., 2023), women can progress from initial opioid misuse to OUD faster than men (i.e., “telescoping”) (Towers et al., 2023; Hernandez-Avila et al., 2004; Back et al., 2011). Some studies have also reported greater opioid craving or relapse in women than men (Back et al., 2011; Maehira et al., 2013), although other studies have reported opposite effects (Nicolas et al., 2022; Gordon et al., 2017; Darke et al., 2007) or no sex differences (Nicolas et al., 2022; Kamal et al., 2007; Kennedy et al., 2013) in these outcomes.

Animal models provide several advantages over human studies (e.g., ability to evaluate the OUD-related effects of opioids in the absence of comorbidities or use of other drugs) and may be useful for better understanding the role of sex in OUD vulnerability. A number of studies have reported greater vulnerability of females than males to the reinforcing effects of opioids using i.v. self-administration (SA) models (e.g., Cicero et al., 2003; Lynch and Carroll, 1999; Smethells et al., 2020; Guha et al., 2022; Towers et al., 2022), which have considerable translational potential because they involve voluntary opioid taking as occurs in humans (Swain et al., 2021). However, some preclinical opioid SA studies have found greater vulnerability in males under at least some conditions (Mavrikaki et al., 2017; Townsend et al., 2019), whereas others have found no sex differences for most or all opioid SA outcomes studied (e.g., Mavrikaki et al., 2017; Stewart et al., 1996; Venniro et al., 2017; Fredriksson et al., 2020). Factors that may contribute to these differences across studies include the opioid used (e.g., morphine versus oxycodone), the phase of OUD modeled (e.g., acquisition versus relapse), or other procedural variables (e.g., schedule of reinforcement). Regardless, these preclinical data parallel the mixed findings regarding sex differences in OUD vulnerability in humans, and emphasize the need for more work in this area.

Behavioral economics, which applies classic economic principles to the experimental analysis of behavior, provides a translationally relevant framework for evaluating the role of sex in OUD vulnerability. Behavioral economics quantifies the change in the consumption of a reinforcer (e.g., opioid) as a function of its unit price, which in drug SA models is operationalized as the cost-benefit ratio of response requirement/unit dose (Hursh, 1991; Bickel et al., 2000; Hursh and Silberberg, 2008). A more rapid decrease in consumption following increases in unit price (greater elasticity of demand) indicates lower abuse liability, while a slower decrease (lower elasticity of demand) indicates greater abuse liability (Hursh, 1991; Bickel et al., 2000; Hursh and Silberberg, 2008). Behavioral economics has served as a sensitive measure of the reinforcing efficacy of opioids and other drugs in both humans and animals (e.g., Aston et al., 2017; Mackillop et al., 2009; Pickover et al., 2016; LeSage et al., 2016; Bentzley et al., 2014), and has been used to study sex differences in the efficacy of several reinforcers including nicotine, cocaine, and food in rodents (Grebenstein et al., 2013; Kohtz et al., 2022; Freeman et al., 2021). However, behavioral economics has been used to only a limited extent to examine sex differences in the preclinical opioid literature, with one study (Townsend et al., 2019) reporting lower elasticity of demand for fentanyl in females and another (Lacy et al., 2020) reporting no overall sex difference in demand for remifentanil.

The goal of this study was to compare elasticity of demand for morphine (0.4 mg/kg/infusion) in an i.v. SA model in male and female rats. We used morphine because it is the prototypical opioid and the primary active metabolite of heroin, and because sex differences have been reported on some measures of morphine SA (e.g., acquisition) (Cicero et al., 2003; Mayberry et al., 2022). Rate of acquisition of morphine SA, predictors of individual differences in demand (e.g., cumulative morphine infusions during acquisition), and relationships between different behavioral economic outcomes (e.g., *α* and *Q*_0_, see below) were also compared across sexes. Such correlational analyses were of interest because sex differences in correlations between measures of opioid SA can be detected even in the absence of sex differences in the SA measures themselves (Guha et al., 2022). We used larger group sizes than are typically used in preclinical studies to facilitate detection of both individual and sex differences in these outcomes, as well as to avoid spurious outcomes that can occur with smaller sample sizes (Szucs and Ioannidis, 2020). Finally, opioid SA can reduce body weight (Chen et al., 2006; Le et al., 2014), a putative marker of physical dependence (Chen et al., 2006). Effects of morphine SA on body weight in both sexes were therefore also examined.

## Methods

### Animals

Experimentally naïve male and female adult Sprague Dawley rats (Inotiv, West Lafayette, IN) weighing 275–300 g (male) or 200–250 g (female) at arrival were used. All rats were individually housed in a temperature- and humidity-controlled colony room with unlimited access to water under a reversed 12-h light/dark cycle. All behavioral testing occurred during the dark (active) phase. Beginning 1 week following arrival, food was restricted to 16 (females) or 18 (males) g/day to facilitate operant performance, avoid detrimental health effects of long-term *ad libitum* feeding, and limit catheter migration. All procedures were approved by the Institutional Animal Care and Use Committee (IACUC) of the Hennepin Health Research Institute in accordance with the NIH Guide for the Care and Use of Laboratory Animals and the Guidelines for the Care and Use of Mammals in Neuroscience and Behavioral Research.

### Drugs

Morphine sulfate (NIH National Institute on Drug Abuse Drug Supply Program, Bethesda, MD) was dissolved in sterile saline and heparin (30 units/mL) was added to maintain catheter patency. The morphine solution was sterile-filtered and its pH was adjusted to 7.4 prior to use. Morphine doses are expressed as the weight of the salt.

### Apparatus

Self-administration (SA) sessions were conducted using standard operant conditioning chambers (model ENV-007, Med Associates, Inc.). Each chamber contained two response levers, a green light emitting diode (LED) cue light located 2 cm above each lever, and a house light that provided ambient illumination. Each chamber was placed inside a sound-attenuating cubicle equipped with an exhaust fan that provided masking noise. An infusion pump (model PHM-100-15, Med Associates) placed outside each cubicle delivered infusions in a volume of 0.1 mL/kg over approximately 1 s. MED-PC IV or V software (Med Associates) was used for operating the experimental apparatus and recording data.

### Surgery

Each rat was implanted with a chronic indwelling catheter into the right jugular vein under isoflurane (1–3%) anesthesia, using general surgical procedures described in detail elsewhere (Harris et al., 2008; LeSage et al., 2002). The catheter was externalized between the scapulae and attached to either a vascular-access harness (VAH95AB, Instech Laboratories, Plymouth Meeting, PA) or an indwelling vascular-access button (VABR2B, Instech Laboratories). In either case, the catheter was connected mid-scapulae to a tether that ran through a fluid swivel before connecting to the drug pump. Immediately following surgery, rats were administered extended-release meloxicam (4 mg/kg, s.c.) for analgesia. Animals were allowed to recover for 1 week after surgery. During the first 3 days of recovery, they received daily i.v. infusions of heparinized saline and ceftriaxone antibiotic (5.25 mg). Infusions of methohexital (0.1 mL, 10 mg/mL, i.v.) were administered to check catheter patency post-session on Fridays. If a catheter became occluded (indicated by a failure of the animal to exhibit anesthesia within 3–5 s after methohexital infusion), another catheter was implanted into a femoral vein. If that catheter failed, a third catheter was implanted into the contralateral femoral vein. Failure of the third catheter resulted in removal of the animal from the study.

### Protocol

Beginning ≈7 days after catheter implantation, rats (*n* = 61, 30 males, 31 females) were allowed to respond for i.v. infusions of morphine sulfate (0.4 mg/kg/infusion) during daily 2-h sessions conducted 7 days per week using our standard apparatus and procedures (Swain et al., 2020; Swain et al., 2018). This unit dose and access duration support reliable morphine SA without inducing the self-mutilation that can occur with higher unit doses and longer sessions (Swain et al., 2018). Responding on the “active” response lever resulted in an i.v. infusion of morphine accompanied by offset of the cue light above the active response lever. Following a 5-s timeout, the cue light above the active lever was illuminated to signal availability of the next infusion. Responses on the other (“inactive”) response lever were recorded but had no programmed consequences. All rats were weighed immediately prior to each daily SA session throughout the protocol. On the first day of morphine SA, food powder was placed on the active lever to facilitate contact with the drug contingency. Data from this session were not included in the data analysis. Rats were tested under a fixed ratio (FR) 1 schedule for at least 10 sessions and until acquisition criteria were met (≥5 infusions per session and a ≥2:1 response ratio on the active versus inactive lever for at least 5 sessions, with no apparent trend), at which point the FR response requirement was increased to FR 2 for at least 5 sessions and until the same criteria were again met. The FR was then increased to FR 3 for at least 10 sessions and until acquisition criteria were met (same criteria as at FR 1 and FR 2, as well as a coefficient of variation ≤20% across 5 sessions). To measure demand, the FR requirement was increased each day as follows: FR 1, 2, 3, 6, 12, 24, 48, 96. We increased unit price *across* sessions rather than *within* each session to account for morphine’s relatively long elimination half-life compared to other opioids (e.g., fentanyl, remifentanil) (e.g., Mullis et al., 1979; Hug and Murphy, 1981). Within-session approaches for increasing unit price are best-suited for drugs with relatively short half-lives, which minimizes effects of prior drug infusions on responding for unit prices later in the session (Lenoir and Ahmed, 2008; Oleson et al., 2011). We found that morphine consumption under this protocol in males at a lower unit dose (0.2 mg/kg/infusion) was well described by the current exponential demand function (Swain et al., 2020; Swain et al., 2018). A negative control group (*n* = 38, 20 males, 18 females) was allowed to respond for i.v. infusions of saline. Because rats do not reliably self-administer saline, increases in FR during “acquisition” for these animals were not based on SA performance. Rather, they occurred on the same day as for a control-paired rat of the same sex from the morphine SA group that began the protocol at a similar time. Rats in the saline group did not acquire stable SA under the FR 3 schedule and therefore were not tested for demand.

### Statistics

Infusions earned during each session during the first 10 acquisition sessions under the FR 1 schedule, the first 5 sessions at FR 2, the first 5 sessions at FR 3, and the final 5 sessions at FR 3 prior to demand testing were compared using separate 3-factor ANOVAs with group (i.e., morphine or saline) and sex as between-subject factors and session as a within-subject factor, followed as appropriate by Bonferroni *post hoc* tests. Active and inactive lever presses during acquisition were analyzed separately in the same manner as a secondary outcome. The number of sessions needed to acquire morphine SA under the FR 1 schedule as defined above were log-transformed because they were not normally distributed and subsequently compared between sexes using an independent samples *t*-test with Welch’s correction to account for unequal variances. Average body weights (in g) during the four acquisition phases described above (first 10 sessions at FR 1, etc.) were analyzed using separate 2-factor ANOVAs with group and sex as between-subject factors, followed when appropriate by Bonferonni *post hoc* tests comparing the morphine and saline group for each sex. Degrees of freedom for all ANOVAs were adjusted using the Greenhouse–Geisser correction to account for possible violations of sphericity. Data for animals lost to attrition during acquisition (see below) were included in analyses for those acquisition phases that they successfully completed.

Infusions at each FR during demand testing in the morphine SA group were compared using a two-factor ANOVA with sex as a between subject factor and FR requirement as a within-subject factor, followed by Dunnett’s *post hoc* tests comparing infusions at FR 1 to those at subsequent FRs. Total active and inactive lever responses at each FR during demand testing were analyzed in the same manner, except that lever (active versus inactive) was included as an additional within-subject variable. To determine elasticity of demand (reinforcing efficacy) during FR escalation, an exponential demand curve analysis was conducted using the following equation:


$$\log\;Q=\log Q_{0}+k\left(e{-}^{\alpha \cdot Q_{0}\cdot C}-1\right)$$

In this model, the quantity consumed (*Q*) of a reinforcer is plotted as a function of its unit price (FR/unit dose). The free parameters, *Q*_0_ and *α* are estimated from the best-fit function and refer to the theoretical maximum level of consumption at zero price (i.e., level or “intensity” of demand) and the rate of change in consumption with increases in unit price (elasticity of demand), respectively. The *k* parameter is a constant specifying the range of consumption in log units (2.8 in the current dataset) that serves to normalize the free parameters across subjects and allow meaningful statistical comparisons between groups. The *k* value is held constant across all data sets being compared, because changes in *k* impact the value of *α*. The *α* parameter is considered a measure of reinforcing efficacy, such that rapidly declining (elastic) demand curves have higher *α* values and indicate lower reinforcing efficacy compared to slower declining (inelastic) demand curves. Because 0 is undefined on a log scale, 0 values in consumption were replaced with 0.04 (1/10th of our lowest non-zero consumption level) to provide better curve fits and more accurate parameter estimates of demand for individual rats (Swain et al., 2020; Koffarnus et al., 2015). Other demand measures of interest included: *Q*_0_, the level or intensity of demand as described above; *P*_max_, or the unit price at which maximal response output occurred; and *O*_max_, or the maximal response output. *P*_max_ and *O*_max_ were determined based on their observed rather than their estimated values. These behavioral economic measures were compared between sexes using independent samples *t*-tests.

Relationships between morphine SA outcomes in each sex were analyzed using linear regression. Outcomes of interest included the behavioral economic measures defined above (i.e., *α*, *Q*_0_, *P*_max_, and *O*_max_), the number of sessions needed to reach acquisition criteria at FR 1, average infusions during the first 10 days of acquisition at FR 1, average infusions during the first 5 days at FR 2, average infusions during the first and final 5 days at FR 3, cumulative number of infusions earned prior to demand testing, and average number of inactive lever presses during the FR 2 and FR 3 acquisition phases (i.e., those acquisition phases in which inactive lever pressing was higher in the morphine compared to the saline groups, see Results) as a measure of morphine’s locomotor stimulant effects. All outcomes used for regression analyses except *α* were log-transformed prior to analysis because they were not normally distributed. Slopes for some linear regression analyses were compared between males and females using an F test for equal slopes. All statistical analyses were performed using GraphPad Prism 10, with significance level set at *α* = 0.05 for all tests.

## Results

### Acquisition

#### Infusions

**Figure 1:**
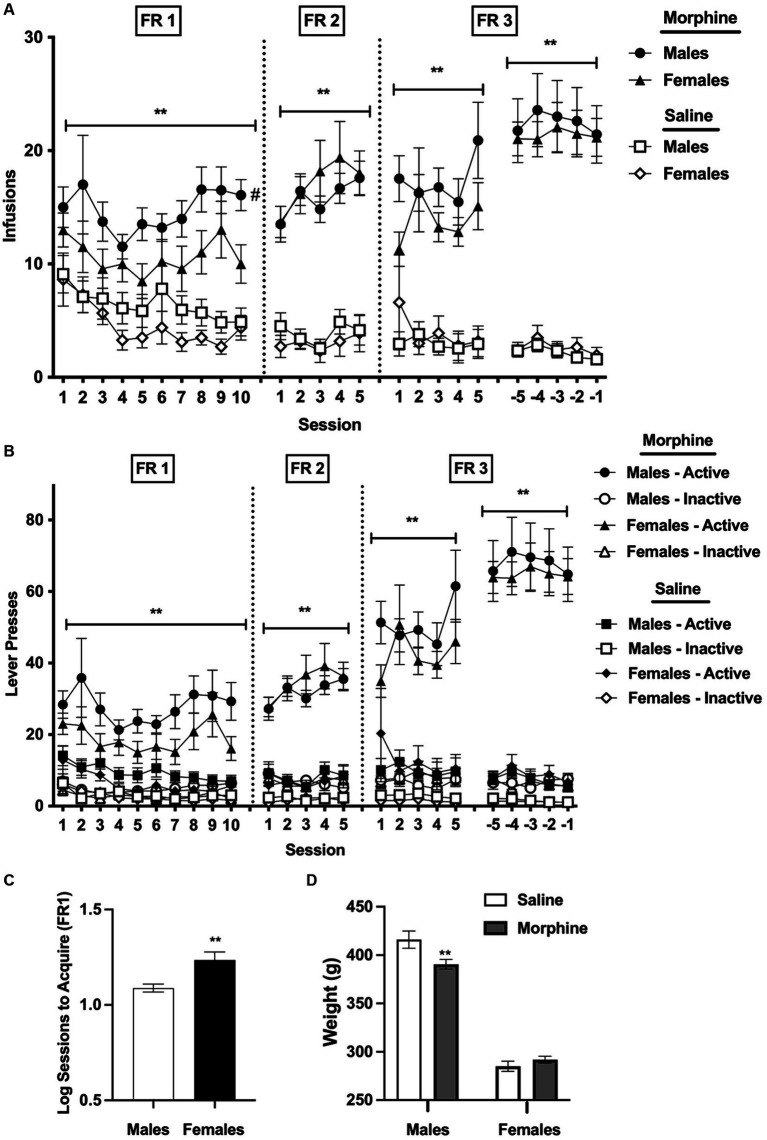
**(A)** Mean ± SEM infusions earned per session **(A)** and active/inactive lever responses **(B)** during acquisition for males and females responding for i.v. morphine (0.4 mg/kg/infusion) or saline. ^**^Significant main effect of group (i.e., morphine versus saline) at that FR, *p* < 0.01. ^#^Male morphine group different from female morphine group at that FR, *p* < 0.05. **(C)** Mean ± SEM sessions required to reach acquisition criteria at FR 1 (log values) in the male and female morphine groups. ^**^Significantly different from males, *p* = 0.01. **(D)** Mean ± SEM weight (in g) for males and females in the morphine and saline groups during the last 5 sessions at FR 3. ^**^Significantly different from saline for that sex, *p* < 0.01.

Analysis of infusions earned per session during the first 10 sessions under the FR 1 schedule indicated a significant main effect of group (i.e., morphine versus saline), [*F*(1, 95) = 26.7, *p* < 0.0001] and sex, [*F*(1, 95) = 4.4, *p* < 0.05], but no significant effects of session or interaction between these variables (Figure 1A). Average infusions across all 10 sessions were significantly higher in the morphine group compared to the saline group for both sexes (*t* = 3.1 and 4.2 for males and females, respectively, *p* < 0.01). Bonferroni post-hoc comparisons, which retain their validity despite the absence of a significant interaction between group and sex in the overall ANOVA (see Howell, 2020), indicated that infusions across all 10 sessions at FR 1 were higher in the male morphine group than in the female morphine group (*t* = 2.3, *p* < 0.05). In contrast, infusions for the male saline group and female saline group did not differ (Figure 1A).

There was a significant main effect of group on infusions during the first 5 sessions at FR 2, [*F*(1, 95) = 92.4, *p* < 0.0001], the first 5 sessions at FR 3, [*F*(1, 92) = 84.0, *p* < 0.0001], and the final 5 sessions at [FR 3, *F*(1, 90) = 91.0, *p* < 0.0001], reflecting higher infusions in the male and female morphine groups compared to the male and female saline groups (Figure 1A). There was also a significant effect of session during the final 5 sessions at [FR 3, *F*(3.3, 300.3) = 2.7, *p* < 0.05]. There were no other significant main effects or interactions at either FR 2 or FR 3.

#### Lever presses

Analysis of active lever presses under the FR 1 schedule indicated a significant main effect of group at FR 1, [*F*(1, 95) = 21.8, *p* < 0.0001], reflecting higher levels of active lever pressing in the morphine compared to the saline group for both sexes (Figure 1B), and a trend toward a main effect of sex, [*F*(1, 95) = 3.3, *p =* 0.07]. There were also significant effects of group during the first 5 sessions at FR 2 [*F*(1, 95) = 92.6, *p* < 0.0001], the first 5 sessions at FR 3 [*F*(1, 94) = 53.7, *p* < 0.0001], and the final 5 sessions at FR 3, [*F*(1, 90) = 86.8, *p* < 0.0001], reflecting continued higher active lever pressing in the morphine compared to the saline groups during these sessions, as well as a significant main effect of session during the final 5 sessions at FR 3, [*F*(3.3, 297.5) = 2.8, *p* < 0.05]. There were no other significant main effects or interactions for active lever presses during acquisition.

Analysis of inactive lever presses indicated a significant main effect of session at FR 1, [*F*(2.7, 252.6) = 3.2, *p* < 0.05], but no effects of group, sex, or interaction between these variables (Figure 1B). There were significant main effects of group at FR 2, [*F*(1, 95) = 19.7, *p* < 0.001], the first 5 sessions at FR 3, [*F*(1, 94.0) = 11.1, *p* < 0.01], and the final 5 sessions at FR 3, [*F*(1, 90) = 16.2, *p* < 0.01], reflecting slightly higher inactive lever pressing in the male and female morphine groups compared to the male and female saline groups (Figure 1B), but no effects of session, sex, or interactions.

#### Sessions to acquire under the FR 1 schedule

Females required significantly more sessions than males to achieve acquisition criteria under the FR 1 schedule (mean ± SEM sessions to acquire in males and females = 12.7 ± 0.7 and 20.0 ± 2.3 sessions, respectively; *t* = 3.2, *p* < 0.01; Figure 1C).

#### Body weights

Body weights did not differ between the morphine and saline group for either males or females under the FR 1 schedule, FR 2 schedule, or the first 5 sessions under the FR 3 schedule (data not shown). Analysis of body weights averaged across the final 5 sessions at FR 3 indicated no significant effect of group, but there was a significant effect of sex, [*F*(1, 90) = 400.0, *p* < 0.0001], and a significant interaction between sex and group, [*F*(1, 90) = 7.9, *p* < 0.01]. Body weights were lower in the morphine group compared to the saline group in males (*t* = 3.2, *p* < 0.01; Figure 1D), but did not differ between the morphine and saline group in females (Figure 1D).

#### Demand

**Figure 2:**
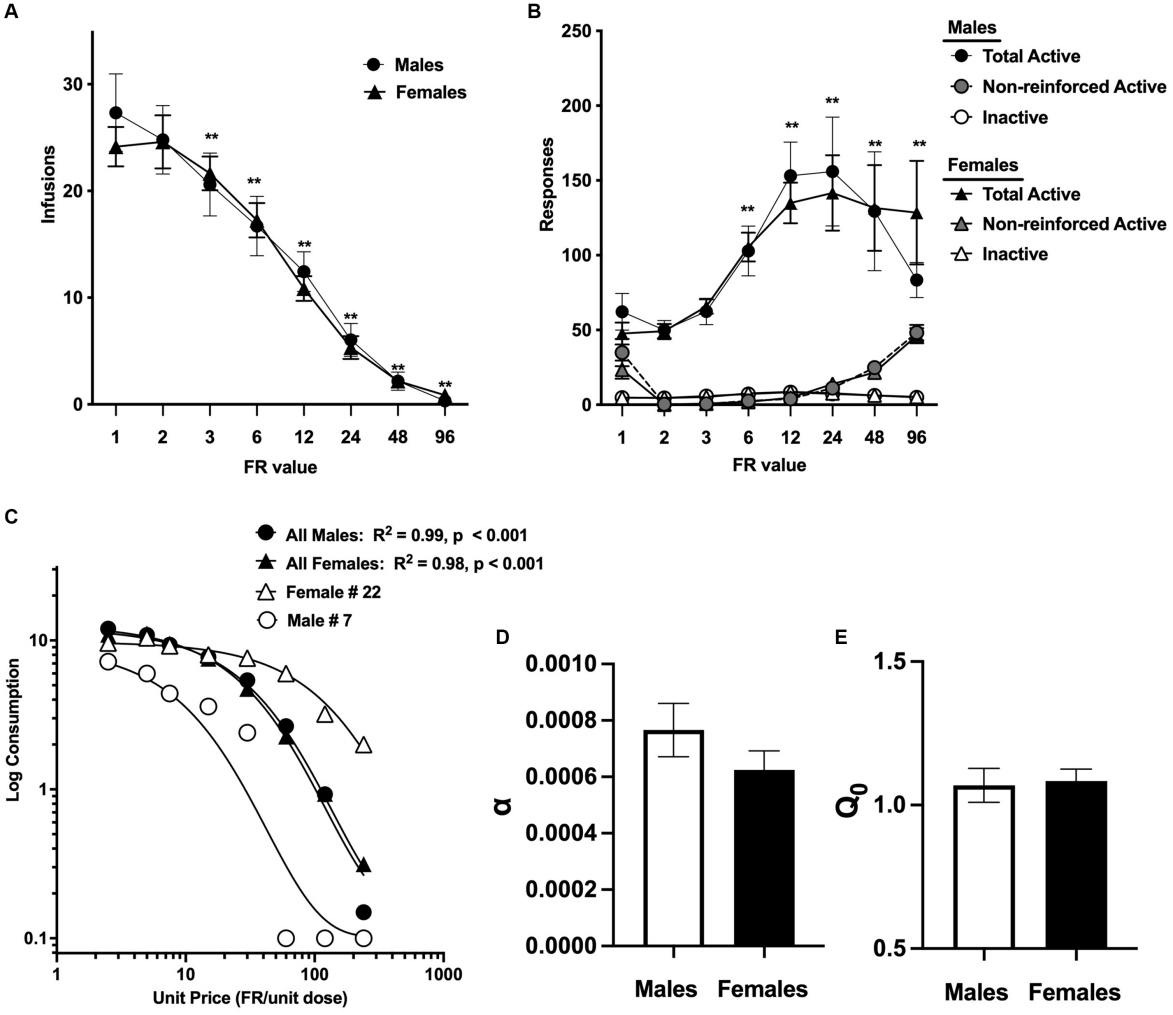
Mean ± SEM infusions earned per session **(A)** and total active lever responses, non-reinforced active lever responses (i.e., active lever responses during the 5 s timeout period), and inactive lever responses **(B)** at each FR during demand testing in the male and female morphine groups. ^**^Significantly different compared to infusions or active lever responses at FR1 (collapsed across sexes), *p* < 0.01. **(C)** Exponential demand curve describing morphine consumption as a function of unit price for rats as a group, and for individual rats with relatively low (female #22) and high (male #7) elasticity of demand (*α*). Mean ± SEM *α* values **(D)** and log-transformed *Q*_0_ values **(E)** in the male and female morphine groups.

A total of 6 rats (3/sex) in the morphine group were lost to attrition during acquisition due to catheter issues, illness, or other problems. Analysis of infusions during FR escalation in the remaining 56 rats (28/sex) indicated a significant effect of FR value, [*F* (2.4, 128.6) = 97.9, *p* < 0.0001], but no effect of sex or FR × sex interaction. Comparison of data collapsed across sex indicated that infusions were decreased compared to FR 1 at all FR values ≥ FR 3 (Dunnett *q* = 4.5–12.3, *p* < 0.01; Figure 2A). Analysis of total active and inactive responses indicated a significant effect of FR value [*F* (7, 378) = 11.8, *p* < 0.0001] and lever (active versus inactive) [*F* (0.4, 23.0) = 86.0, *p* < 0.0001], as well as a significant interaction between FR value and lever [*F* (3.1, 163.7) = 11.3, *p* < 0.0001]. There was no significant effect of sex and no significant interactions related to sex. Comparison of data collapsed across sex indicated that total active lever responses were increased compared to FR 1 at all FR values ≥FR 6 (*q* = 3.0–8.8, *p* < 0.05 or 0.01; Figure 2A). The similar number of infusions at FR 1 and FR 2 in both sexes (Figure 2A) suggests that total active lever responses should double between these FRs, yet this was not the case for either sex (Figure 2B). This reflects the higher levels of non-reinforced active lever responses during the 5 s timeout at FR 1 compared to FR 2 in both sexes (Figure 2B, grey symbols).

**Figure 3:**
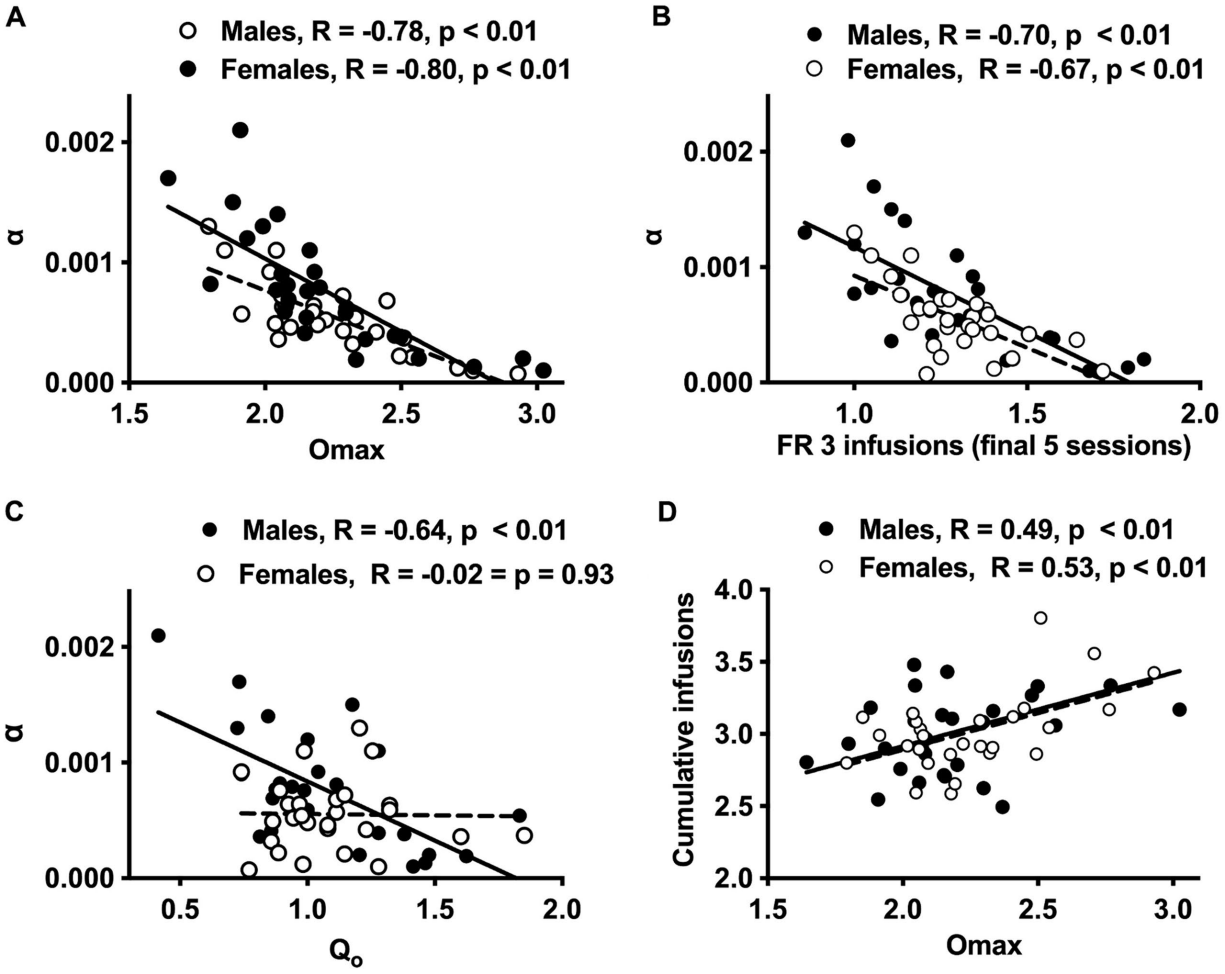
Scatterplots with regression line depicting the relationship between *O*_max_ and *α* **(A)**, infusions during the final 5 sessions at FR 3 and *α* **(B)**, *Q*_0_ and *α* **(C)**, and *O*_max_ and cumulative morphine infusions earned prior to demand testing **(D)** in males and females. Regression lines are solid for males and dashed for females. Lower *α* values = greater reinforcement efficacy.

Morphine consumption during demand testing was well described by an exponential demand function for both males and females, with *R*^2^ values ≥0.85 for the majority of rats of each sex (Table 1) and *R*^2^ = 0.98 or 0.99 for females and males as a group, respectively (Figure 2C). There was considerable individual variability in *α* values (i.e., elasticity of demand) in both sexes, with some rats showing a rapid decline in morphine consumption following increases in FR (e.g., male #7 in Figure 2C) and others maintaining significant consumption despite the increases in unit price (e.g., female #22 in Figure 2C). There was a 21.0-fold and 18.1-fold range in *α* values across individual males and females, respectively (Table 1; see also scatterplots of *α* values shown in Figures 3A–C). There were no sex differences in *α* (Figure 2D), *Q*_0_ (Figure 2E), *P*_max_, or *O*_max_ (Table 1).

**Table 1:** Exponential demand curve parameters for individual subjects.

Subject	*α*	*Q*_0_	*P*_max_	*O*_max_	*R* ^2^
**Males**					
1	0.00092	11.0	30	152	0.92
2	0.00077	7.5	30	110	0.96
3	0.00041	7.2	60	140	0.94
4	0.00013	29.0	30	587	0.83
5	0.00069	7.3	60	122	0.93
6	0.00130	5.3	60	98	0.89
7	0.00150	15.0	30	76	0.91
8	0.00082	7.8	15	63	0.98
9	0.00170	5.4	120	44	0.94
10	0.00079	8.7	30	159	0.94
11	0.00062	9.2	60	199	0.95
12	0.00090	5.5	60	115	0.96
14	0.00020	16	60	367	0.99
15	0.00059	10	30	118	0.99
16	0.00038	24	30	315	0.97
17	0.00120	10	30	86	0.96
19	0.00210	2.6	60	81	0.77
20	0.00020	30	60	888	0.80
21	0.00039	19	60	300	0.95
22	0.00054	68	30	142	0.61
23	0.00019	42	60	216	0.91
24	0.00110	19	30	146	0.88
25	0.00058	10	60	198	0.95
26	0.00140	7	30	111	0.90
28	0.00010	26	120	1,056	0.92
29	0.00076	9.7	30	143	0.95
30	0.00081	13	30	121	0.92
31	0.00036	6.5	120	234	0.96
**Mean**	**0.000766**	**15.4**	**50.9**	**228.1**	**0.91**
**SEM**	**0.000094**	**2.6**	**5.4**	**45.2**	**0.02**
**Females**					
1	0.00058	8.8	120	167	0.92
2	0.00120	9.8	30	71	0.97
3	0.00130	15.0	30	110	0.90
4	0.00011	19.0	120	580	0.94
5	0.00046	17	60	256	0.90
7	0.00100	5.6	30	104	0.95
8	0.00084	8.0	30	112	0.95
9	0.00073	20.0	7.5	116	0.97
10	0.00064	13.0	60	82	0.93
12	0.00071	8.5	30	151	0.89
13	0.00023	14.0	60	348	0.99
14	0.00076	13.0	60	281	0.86
15	0.00071	9.4	30	119	0.98
16	0.00042	35	30	112	0.94
17	0.00160	14	15	62	0.92
18	0.00082	13	30	193	0.92
19	0.00082	13	30	115	0.95
20	0.00053	10	240	156	0.92
21	0.00034	7.2	120	210	0.97
22	0.00013	9.6	240	511	0.97
23	0.00047	12	60	194	0.94
24	0.00023	7.7	120	312	0.96
25	0.00054	7.8	15	109	0.71
26	0.00051	12	60	124	0.95
27	0.00060	9.4	60	215	0.95
28	0.00045	57	30	324	0.77
29	0.00069	20	15	150	0.96
30	0.000077	5.9	240	851	0.73
**Mean**	**0.000625**	**14.1**	**70.5**	**219.1**	**0.92**
**SEM**	**0.000067**	**1.9**	**12.9**	**33.3**	**0.01**

#### Correlates

Higher *O*_max_ values (maximal response output) and a higher number of infusions earned during the FR 2 and FR 3 phases of acquisition were associated with lower *α* values (greater reinforcing efficacy) in both sexes (Figures 3A,B and Table 2). In contrast, higher *Q*_0_ values were associated with lower *α* values in males but not females (Figure 3C), while higher *P*_max_ values were associated with lower *α* values in females but not males (Table 2). Several other relationships between morphine SA measures were similar in both sexes, whereas others were sex-specific (Table 2). For example, *O*_max_ values were significantly correlated with infusions earned during the FR 2 and FR 3 phases and cumulative morphine infusions earned prior to demand testing in both sexes (Table 2 and Figure 3D), while *O*_max_ was associated with *Q*_0_ in males but not females (Table 2).

**Table 2:** Correlation (Pearson’s *R*) between various measures of morphine SA in males (top panel) and females (bottom panel).

Variables	*α*	*Q*_0_	*P*_max_	*O*_max_	Acquire	FR1	FR2	FR3 (first 5)	FR3 (final 5)	Cumulative
**Males**										
*α*										
*Q*_0_	**−0.64** ^**^									
*P*_max_	−0.12	−0.15								
*O*_max_	**−0.78** ^**^	**0.60** ^**^	0.29							
Acquire	0.02	0.03	0.24	−0.09						
FR1	−0.10	0.15	0.03	0.15	**−0.58** ^**^					
FR2	**−0.43** ^*^	0.33	0.24	**0.42** ^*^	−0.28	**0.38** ^*^				
FR3 (first 5)	**−0.51** ^**^	**0.39** ^*^	**0.28** ^*^	**0.63** ^**^	−0.19	0.27	**0.60** ^**^			
FR3 (final 5)	**−0.73** ^**^	**0.74** ^**^	0.09	**0.87** ^**^	0.02	0.10	**0.48** ^**^	**0.66** ^**^		
Cumulative	−0.33	**0.48** ^*^	−0.17	**0.49** ^**^	−0.09	0.16	**0.40** ^*^	0.22	**0.60** ^**^	
Inactive	0.22	−0.08	−0.27	**−0.40** ^*^	0.19	−0.12	−0.32	**−0.40** ^*^	−0.31	−0.05
**Females**										
*α*	—									
*Q*_0_	−0.02									
*P*_max_	**−0.62** ^**^	−0.35	—							
*O*_max_	**−0.80** ^**^	−0.01	**0.67** ^**^	—						
Acquire	0.21	0.03	0.11	0.09	—					
FR1	−0.22	0.04	−0.01	−0.08	**−0.57** ^**^	—				
FR2	**−0.46** ^*^	−0.11	0.36	**0.53** ^**^	−0.13	0.17	—			
FR3 (first 5)	**−0.57** ^**^	0.02	0.35	**0.57** ^**^	−0.15	0.19	**0.87** ^**^	—		
FR3 (final 5)	**−0.67** ^**^	**0.56** ^**^	0.16	**0.57** ^**^	−0.12	0.28	0.26	**0.54** ^**^		
Cumulative	−0.35	**0.42** ^*^	0.15	**0.53** ^**^	0.29	−0.09	0.32	0.37	**0.50** ^**^	
Inactive	−0.07	0.16	−0.33	−0.08	−0.22	0.25	0.20	0.35	0.31	0.30

*α* = rate of change in consumption with increases in unit price (elasticity of demand). Lower *α* = greater reinforcement efficacy. *Q*_0_ = consumption at zero price (intensity of demand). *P*_max_ = unit price at which maximal response output occurred. *O*_max_ = maximal response output. Acquire = Number of sessions needed to reach acquisition criteria at FR 1 (see Methods). FR 1 = average infusions during first 10 days of acquisition at FR 1. FR 2 = average infusions during the first 5 days at FR 2. FR 3 (first 5) = average infusions during the first 5 days at FR 3. FR 3 (final 5) = average infusions during the final 5 days at FR 3 prior to demand testing. Cumulative: cumulative number of infusions earned prior to demand testing. Inactive: average inactive lever pressing during FR 2 and FR 3 phases. All outcomes except *α* were log-transformed because they were not normally distributed. Bold values indicate statistically significant relationships, *p* < 0.05 (*) or *p* < 0.01 (**).

Analysis of data portrayed in Figures 3A–C indicated a significant difference in the slope of the linear regression lines describing the relationship between *Q*_0_ and *α* values in males versus females [*F* (1, 52) = 7.9, *p* ≤ 0.001; Figure 3C]. In contrast, there were no sex differences in slopes of the regression lines describing the relationships between the other variables (Figures 3A,B,D).

## Discussion

The main finding of this study was that elasticity of demand in an i.v. morphine SA model did not differ between male and female rats. Most predictors of individual differences in demand also generalized across sexes. Acquisition of morphine SA under a FR 1 schedule of reinforcement was slower and infusions earned were lower in females than in males, but morphine SA did not differ between sexes during the subsequent FR 2 and FR 3 phases of acquisition.

Our data are consistent with findings of similar OUD vulnerability in males and females in some human and animal studies (e.g., Nicolas et al., 2022; Kennedy et al., 2013; Stewart et al., 1996) Nonetheless, we acknowledge that a number of studies have demonstrated clear sex differences in OUD vulnerability in both species (e.g., Hernandez-Avila et al., 2004; Lynch and Carroll, 1999; Townsend et al., 2019). Taken together, these studies suggest that factors mediating the relationship between sex and OUD vulnerability may be complex and only apparent under certain conditions.

The factor(s) contributing to differences in our findings versus other preclinical studies that found sex differences in i.v. opioid SA are not clear. It is unlikely that this discrepancy is due to our study being underpowered to detect sex differences, as our group sizes were actually similar to or larger than those often used in this literature. It also is not attributable to our use of morphine, as two prior studies have reported differences in i.v SA of this opioid (Cicero et al., 2003; Mayberry et al., 2022). Specifically, Mayberry et al. (2022) reported that females earned a higher number of morphine infusions than males during a 10 day acquisition period under a FR 1 schedule, which is opposite of the current findings, although there were no sex differences of incubation of morphine-seeking in that study (Mayberry et al., 2022). Furthermore, Cicero et al. (2003) reported greater reinforcing efficacy (i.e., higher breakpoints) in females than males responding for i.v. morphine under a progressive ratio (PR) schedule of reinforcement. Methodological factors that could account for the difference between our findings and those of Mayberry et al. (2022) include rat strain (Sprague Dawley in this study versus Long Evans in the Mayberry study), training dose (0.4 mg/kg/infusion versus 0.75 mg/kg/infusion), and duration of morphine SA access (2 h/day versus 12 h/day), among others. Although the Cicero et al. (2003) study used the same rat strain and vendor/source as our study, it differed in other respects including its use of a longer (4 h/day) access schedule and a considerably different acquisition procedure in which rats were first trained to respond for food, then switched to i.v. heroin SA, and then switched to morphine SA. Perhaps more importantly, the morphine unit dose in Cicero et al. (2003) was 150 μg/infusion regardless of body weight, resulting in females receiving a higher morphine unit dose due to their lower body weight compared to males (e.g., ≈ 0.7 versus 0.4 mg/kg/infusion for ≈ 90-day old females and males, respectively). This may have contributed to the higher breakpoints in females because higher unit doses in drug SA models generally support higher breakpoints (Stafford et al., 1998; Grasing et al., 2003).

This study provided the opportunity to extend a behavioral economic framework to morphine SA in females. Consistent with our previous studies in male rats trained using a lower morphine SA unit dose (Swain et al., 2018; Liu et al., 2021; Swain et al., 2021), an exponential demand function provided an excellent fit for morphine consumption in both sexes, with considerable individual differences in *α*. As such, these data further support the generalizability and utility of behavioral economics for evaluating determinants of OUD vulnerability.

We evaluated predictors of individual differences in elasticity of demand (*α*) that were measured prior to demand testing (e.g., rate of acquisition), as well as correlates derived from demand testing itself (e.g., *Q*_0_). Among the former, higher infusions during the FR 2 and FR 3 acquisition phases predicted lower elasticity of demand (greater reinforcing efficacy) in both sexes. This is consistent with preclinical findings indicating that higher baseline levels of nicotine SA predict lower demand elasticity (Grebenstein et al., 2015). Higher typical (e.g., past-year) levels of consumption of opioids and other drugs also predicts lower demand elasticity in humans (Pickover et al., 2016; Bertholet et al., 2015). Together these findings implicate baseline drug intake as a sensitive prospective indicator of demand. Among the correlates derived from demand testing, *O*_max_ (maximal response output) was closely correlated with elasticity of demand and several other morphine SA outcomes (e.g., infusions during the FR 2 and FR 3 acquisition phases) in both sexes. This close correspondence between *O*_max_ and demand and other measures of drug use has also previously been reported in humans (Murphy et al., 2011; Murphy et al., 2009). Together, these findings support the sensitivity and generalizability of *O*_max_ as a measure of addiction vulnerability in both species.

As is common in this literature (Guha et al., 2022; Townsend et al., 2019; Mayberry et al., 2022) and in line with the suggestion that estrous cycle monitoring is not essential when first evaluating sex differences in a preclinical model (Becker and Koob, 2016), we did not track the estrous cycle. Nonetheless, given that sex hormones can influence SA of opioids and other drugs (e.g., Towers et al., 2022; Becker et al., 2017; Lopresti et al., 2020), we cannot rule out the possibility that sex differences could have been detected had we analyzed data according to estrous phase. This seems unlikely, however, as any increases in morphine SA in females compared to males during certain phases of the cycle would need to be fully offset by decreases in morphine SA during other phases to yield the almost superimposable mean levels of SA across sexes. This would also result in greater day-to-day variability in morphine SA for females compared to males, which was not observed (e.g., mean coefficient of variation during final 5 sessions at FR 3 for males and females was 12.1 and 12.2%, respectively).

A further potential limitation is that only one morphine SA unit dose was studied. Future studies could evaluate the generality of our findings to other morphine unit doses by increasing unit price during demand testing using unit dose reduction rather than FR escalation. To the extent that these two approaches for increasing unit price produce functionally equivalent effects on drug consumption (Bickel et al., 1990; Smith et al., 2016; DeGrandpre et al., 1993), a unit dose reduction protocol would likely yield similar findings as our FR escalation protocol in terms of morphine’s reinforcing efficacy. Nonetheless, a unit dose reduction protocol would allow analysis of sex differences in morphine’s reinforcing potency (lowest dose that maintains SA).

The reduced weight gain in males during morphine SA is consistent with prior studies (Le et al., 2014; Lee et al., 2017) and may reflect the development of physical dependence, although future studies should measure other signs of dependence (e.g., somatic withdrawal signs such as wet-dog shakes, etc.) to confirm this interpretation. The effect of morphine SA on weight in males was only observed at the end of the FR 3 acquisition period, suggesting that it was a consequence of the higher levels of morphine SA (see Figure 1A) and/or higher cumulative morphine exposure during this phase compared to previous phases. This effect of morphine SA on weight was not due to a reduction in food intake, as all rats finished their daily allotment of food throughout the protocol, although it is unclear what other factors (e.g., increased metabolism, hyperthermia, increased locomotor activity, diarrhea during overnight withdrawal periods) may have contributed. In contrast, morphine SA did not affect weight in females, consistent with findings that repeated noncontingent injections of morphine reduced body weight to a greater extent in males compared to females (Boghossian et al., 2001).

In conclusion, our findings indicate generally similar acquisition, demand, and predictors/correlates of demand in an i.v. morphine SA model in male and female rats. Given that distinct neurobiological mechanisms can mediate addiction-related behavior in males and females even in the absence of sex differences in the behavior itself (Mayberry et al., 2022; Becker and Koob, 2016; Becker et al., 2017), comparison of the mechanisms mediating morphine SA in males and females in this model is warranted. Evaluating genetic, transcriptomic, and epigenetic mechanisms underlying demand in both sexes is of particular interest given the involvement of these mechanisms in individual differences in SA of morphine and other opioids (Ambrosio et al., 1995; Browne et al., 2023; Browne et al., 2020). Indeed, the minimal sex differences in morphine SA in this model could be an advantage for this purpose, as the mechanisms underlying morphine SA in males and females could be compared in the absence of sex differences in opioid intake that could complicate data interpretation.

## Data Availability

The original contributions presented in the study are included in the article/supplementary material, further inquiries can be directed to the corresponding author.
